# Supplementary material for: A Personalized Predictive Model That Jointly Optimizes Discrimination and Calibration
Source: Stat Med. 2025 May 16;44(10-12):e70077. doi: 10.1002/sim.70077 (PMC12083855; doi:10.1002/sim.70077)
Supplement: Supplementary file 1 — Data S1. [file SIM-44-0-s001.pdf]

Supplementary Material for A Personalized Predictive Model that Jointly Optimizes Discrimination and Calibration

by Tatiana Krikella and Joel A. Dubin

1 Table S1

α	Proportion	AUROC	CITL	Slope	ICI
0.475	0.0694	**0.9194** (0.011) (0.8945, 0.9264)	**0.0273** (0.015) (0.0046, 0.0694)	**0.8519** (0.141) (0.7437, 1.058)	**0.0276** (0.011) (0.0104, 0.0526)
0.49	0.0694	**0.9194** (0.011) (0.8945, 0.9264)	**0.0273** (0.015) (0.0046, 0.0694)	**0.8519** (0.141) (0.7437, 1.058)	**0.0276** (0.011) (0.0104, 0.0526)
0.5	0.0781	**0.9226** (0.010) (0.8993, 0.9290)	**0.0315** (0.015) (0.0084, 0.0702)	**0.9473** (0.131) (0.8291, 1.1786)	**0.0291** (0.012) (0.0120, 0.0629)
0.58	0.1172	**0.9359** (0.009) (0.9064, 0.9472)	**0.0349** (0.016) (0.0110,0.0752)	**1.226** (0.131) (1.0472, 1.6587)	**0.0331** (0.012) (0.0151, 0.0671)
0.6	0.1215	**0.9381** (0.009) (0.9073, 0.9501)	**0.0357** (0.017) (0.0106, 0.0772)	**1.254** (0.134) (1.089, 1.676)	**0.0347** (0.0121) (0.0169, 0.0677)
0.62	0.1259	**0.9382** (0.009) (0.9074, 0.9501)	**0.0379** (0.016) (0.0144, 0.0796)	**1.278** (0.134) (1.1013, 1.7151)	**0.0377** (0.012) (0.0217, 0.0729)
0.750	0.1563	**0.9438** (0.009) (0.9185, 0.9580)	**0.0426** (0.017) (0.0213, 0.0828)	**1.4325** (0.146) (1.2643, 1.9152)	**0.0461** (0.012) (0.0306, 0.0791)
0.85	0.6424	**0.9223** (0.011) (0.9098, 0.9482)	**0.0474** (0.015) (0.0332, 0.0748)	**1.5337** (0.131) (1.4832, 1.7624)	**0.0581** (0.012) (0.0490, 0.0814)
0.9	0.8594	**0.8688** (0.015) (0.8461, 0.9035)	**0.0196** (0.010) (0.0058, 0.0588)	**1.141** (0.097) (1.099, 1.326)	**0.0240** (0.009) (0.0096, 0.0441)
0.99	0.9983	**0.8614** (0.015) (0.8399, 0.8940)	**0.0189** (0.10) (0.0036, 0.0521)	**1.098** (0.097) (1.076, 1.281)	**0.0229** (0.009) (0.0085, 0.0409)

Table 1: Proportion is optimal proportion, M, found in training step of algorithm. Point estimates are given for each of the performance measures. First set of parentheses contain the bootstrap standard error for the estimates. Second set of parentheses contain the BCa confidence interval.
